# The Reduction of Skin Photodamage by the Ectoine– *Thermus thermophilus* Cofermentation Products

**DOI:** 10.1111/jocd.70298

**Published:** 2025-06-24

**Authors:** Yiyu Wang, Jiayi Liang, Pengkun Zhang, Guihui Yan, Hui Jiang, Meijing Wang

**Affiliations:** ^1^ Hangzhou Sanshi Cosmetics Co. Ltd Hangzhou China; ^2^ College of Life Sciences Zhejiang University Hangzhou China; ^3^ Umotor Biological Technology Co. Ltd Hangzhou China; ^4^ Zhejiang University‐Lishui Joint Innovation Center for Life and Health & Lishui Lvgu Institute for Life and Health Lishui China

**Keywords:** anti‐inflammatory, fibroblasts, microorganisms, photodamage, ultraviolet

## Abstract

**Background:**

Prolonged exposure to UVB (280–320 nm) can lead to skin oxidative damage, inflammatory response, and skin cancer. Many active ingredients in the fermentation products of *Thermus thermophilus* have been shown to play important roles in antioxidant and anti‐UVB photodamage, such as superoxide dismutase (SOD) and photolyase. Ectoine, as one of the most prevalent compatible solutes in halophilic bacteria, can protect cells, proteins, cell membranes, and nucleic acids from external extreme environments such as high temperature, freezing, irradiation, and drying. It has been applied in the industries of fine chemicals, biomedicine, and biomanufacturing worldwide.

**Aims:**

In this study, we evaluated the antioxidant activities and anti‐UVB photodamage activities of Ectoine– *T. thermophilus* cofermentation products (D‐Ectoine).

**Methods:**

The comparison between D‐Ectoine and Ectoine was analyzed with hydroxyl radical (OH·^−^) scavenging assay, superoxide anion (O_2_·^−^) assay, total antioxidant capacity (T‐AOC) assay, and 1,1‐diphenyl‐2‐picrylhydrazyl radical (DPPH) scavenging assay. We evaluated the antioxidant ability of D‐Ectoine by detecting malondialdehyde (MDA) levels: the activity of SOD, catalase (CAT), and glutathione peroxidase (GSH‐PX) in human skin fibroblast (HSF) cells. Inflammatory response was measured with lipopolysaccharides (LPS) induced expression of interleukin 6 (IL‐6) and interleukin 8 (IL‐8). We evaluated the protective ability of D‐Ectoine against UVB‐induced damage by detecting the content of collagen type I (Col I) and extracellular matrix metalloproteinase (MMP‐1) in the cells, and the number of cell survival, erythroid 2‐related factor 2 (Nrf2) and 8‐hydroxy‐2′‐deoxyguanosine (8‐OHdG) expression in 3D skin model.

**Results:**

The results showed that D‐Ectione could promote the GSH‐PX activity in HSF cells with stronger T‐AOC and DPPH scavenging capacity, reduce the expression of LPS‐induced inflammatory factors IL‐6 and IL‐8, increase Col I expression both with and without UVB and inhibit expression of MMP‐1 after UVB irradiation. In the 3D skin model, D‐Ectione could increase the number of cell survival and Nrf2 expression, and decrease 8‐OHdG expression after UVB irradiation.

**Conclusions:**

These results demonstrated that D‐Ectione has protective effects against UVB‐induced skin photodamage and may contribute to the development of cosmetic products with anti‐UVB.

**Abbreviations**
3OAA: 3‐O‐ethyl‐l‐ascorbic acid
8‐OHdG: 8‐hydroxy‐2′‐deoxyguanosine
AD: atopic dermatitis
CAT: catalase
Col I: collagen type I
CPD: cyclobutane pyrimidine dimer
ddH_2_O: double‐distilled water
DMSO: dimethyl sulfoxide
DPPH: 1,1‐diphenyl‐2‐picrylhydrazyl
DSS: dextran sulfate sodium
DXM: dexamethasone
ECM: extracellular matrix
GSH‐PX: glutathione peroxidase
GSSG: oxidized glutathione
H&E: hematoxylin and eosin
HaCaT: human immortalized epidermal
HSF: human skin fibroblast
HSF: human skin fibroblasts
Hsp70: heatshock protein 70
IBD: inflammatory bowel disease
ICAM‐1: intercellular adhesion molecule‐1
IL‐6: interleukin 6
IL‐8: interleukin 8
IOD: integrated optical density
LPS: lipopolysaccharide
LPS: lipopolysaccharide
MAPK: mitogen‐activated protein kinase
MDA: malondialdehyde
MMP: matrix metalloproteinase
mtDNA: mitochondrial DNA
MTT: methylthiazolyldiphenyl‐tetrazolium bromide
NF‐κB: nuclear factor‐kappa B
Nrf2: erythroid 2‐related factor 2
O_2_·^−^
: superoxide anion free radical
OH·^−^
: hydroxyl radical
PBS: phosphate buffered saline
PUFA: polyunsaturated fatty acid
ROS: reactive oxygen species
SEM: standard error of the mean
SOD: superoxide dismutase

*T. thermophilus*

:
*Thermus thermophilus*

T‐AOC: total antioxidant capacity
TGF‐β1: transforming growth factor beta1
TNF‐α: tumor necrosis factor‐α
TtSOD: superoxide dismutase
UVA: Ultraviolet radiation A
UVB: Ultraviolet radiation B
UVC: Ultraviolet radiation C
Vc: vitamin C
VE: vitamin E
α‐MSH: α‐melanocyte‐stimulating hormone

## 1 Introduction

Ultraviolet (UV) radiation is ubiquitous in nature and artificial environments, and the skin is the largest and outermost organ of the human body. When exposed to sunlight or UV radiation, it can lead to sunburn, inflammation, photoimmunosuppression, photoaging, and even skin cancer [1]. UV radiation has a wavelength range of 100–400 nm and is categorized into three basic types: UVA (320–400 nm), UVB (280–320 nm), and UVC (100–280 nm). Among them, UVB has been implicated in DNA damage, reactive oxygen species (ROS) production, and extracellular matrix (ECM) alterations, resulting in sunburn and photodamage of the skin. UV‐induced photodamage involves the production of ROS and DNA mutations [2]. Excess ROS activates the mitogen‐activated protein kinase (MAPK) pathway and the nuclear factor‐kappa B (NF‐κB) pathway, promoting the expression of matrix metalloproteinases (MMPs) and inflammatory factors, degrading proteins in the ECM and resulting in apoptosis, cellular senescence, and wrinkles in the skin.

*Thermus thermophilus* is a widespread genus of thermophilic bacteria that can grow and develop at temperatures above 55°C [3]. The *T. thermophilus* fermentant mainly acts as a kind of antioxidant and skin protector under sunlight/UV radiation, which has been used as a cosmetic ingredient for many years in domestic and international cosmetic products [4, 5, 6, 7, 8]. *T. thermophilus* is characterized by fast growth rates, high cell culture yields, efficient expression of natural components, and outstanding thermal stability of proteases, making it a model microorganism for studying the molecular basis of thermophilic bacteria [9]. The genome sequence of the *T. thermophilus* was completed in 2004, which provided the basis for further analysis of its evolutionary mechanism and effective components [10].

The fermentation broth of *T. thermophilus* has been found to stimulate the proliferation of human immortalized epidermal (HaCaT) cells and HSF cells. Additionally, *T. thermophilus* fermentation broth alleviated UVB‐induced photodamage and promoted cell migration in HSF cells [11]. The components of *T. thermophilus*, such as superoxide dismutase (TtSOD) and photolyase, have been found to scavenge ROS, alleviate UVB‐induced cyclobutane pyrimidine dimers (CPDs), and protect cells from surviving in adverse environments. Given that SOD can maintain a dynamic balance between the production and removal of bio‐oxidants in vivo and prevent the toxic effects of free radicals, it has been applied to medicine, food, and cosmetic production [12]. A recombinant TtSOD was purified and characterized after being expressed in *Escherichia coli* , was thermostable at 80°C and 90°C, and retained 57% activity after 60 min at 100°C [13]. Treatment with TtSOD reduced intestinal enlargement and neutrophil infiltration, leading to an attenuation of inflammatory bowel disease (IBD) in zebrafish [14]. In mice, oral administration of TtSOD suppressed intestinal inflammation and maintained intestinal barrier function in dextran sulfate sodium (DSS)‐induced colitis. In addition, TtSOD inhibited LPS‐induced ROS production and inflammatory responses in bone marrow‐derived macrophages. Photolyase from *T. thermophilus* was recombinantly expressed in *E. coli* and demonstrated its highly efficient enzymatic activity in CPD photodamage removal at a low enzyme concentration (15 μg/mL) when DNA was exposed to UV radiation [15]. Based on experiments with mice, wrinkle formation, epidermal hyperplasia, and collagen degradation induced by UVB were significantly inhibited when photolyase was topically applied to the skin. Photolyase reduced UVB‐induced DNA damage, ROS production, and malondialdehyde (MDA) levels; enhanced the activity of SOD, CAT, and GSH‐PX; inhibited the expression of tumor necrosis factor‐α (TNF‐α), interleukin 6 (IL‐6), and IL‐1β in HaCaT cells [16].

1,4,5,6‐Tetrahydro‐2‐methyl‐4‐pyrimidinecarboxylic acid was named ectoine due to its discovery in the halophilic bacterium Ectothiorhodospira by Galinski in 1985 [17]. As a compatible solute, ectoine can bind to water through seven strong hydrogen bonds (H‐bonds) to generate a hydration shell [18]. Ectoine plays an important role as a protectant against various external pressures, such as hyperosmolarity, high temperature, dryness, and radiation in cells [19]. Because of its excellent crucial bioactivities in the stabilization of biomacromolecules, such as proteins, nucleic acids, and cell membranes, ectoine can be utilized in cosmetics and pharmaceutical industries. However, traditional ectoine production in high‐salinity media causes equipment corrosion and an unfriendly environment. To meet its growing commercial demands, heterologous production of ectoine in nonhalophilic microorganisms can have a higher production and a lower cost of ectoine [19, 20].

Ectoine is a very useful skin protectant in cosmetics by prevention of dehydration of skin, recovery of skin barrier function, and prevention of skin aging due to its strong water‐binding activity [21, 22]. Ectoine counteracts the effects of UVA‐induced skin aging through preventing UVA‐induced second messenger release, transcription factor AP‐2 activation, intercellular adhesion molecule‐1 (ICAM‐1) expression, and mitochondrial DNA (mtDNA) mutation in human keratinocytes [23]. Ectoine was able to protect keratinocyte cells from stress conditions by maintaining an elevated level of the heatshock protein 70 (Hsp70), which inhibits genetic expression of proinflammatory cytokines [24]. Interestingly, it is demonstrated that ectoine prevents DNA damage caused by ionizing radiation [25]. It was investigated that ectoine has skin‐whitening effects via inhibition of α‐melanocyte‐stimulating hormone (α‐MSH) stimulated melanogenesis and activation of nuclear factor Nrf2 in UVA‐irradiated HaCaT cells [26]. In addition, when ectoine is applied to the anionic surfactant solution, irritant potential and solubility of model sebum decreased by about 10%–20%, and cell cytotoxicity reduced by up to 60% [27]. A randomized, vehicle‐controlled clinical trial of formulations containing 2% ectoine has shown good antiaging properties in terms of skin hydration, skin elasticity, and skin surface structure than the vehicle treatment [28]. After topical application of an ectoine‐containing cream (EHK02‐01) in patients with mild‐to‐moderate atopic dermatitis (AD), it was found that the efficacy of EHK02‐01 treatment was equivalent to the reference product in a randomized, intra‐individual, double‐blind, multicenter trial [29]. When combined with mannitol, it was demonstrated that this association significantly preserved intracellular glutathione levels, reduced DNA damage induced by ROS, and maintained Langerhans cell functionality in skin cell models [30].

It is known that many of the metabolites of extremophiles have the potential to be valuable resources for the development of a bio‐based economy. Both *T. thermophilus* fermentant and ectoine‐producing halophiles come from extremophiles. Therefore, we speculate that products containing both ectoine and *T. thermophilus* fermentant will have a better protective effect on human skin. In this study, we obtained the Ectoine– *T. thermophilus* cofermentation products (named as D‐Ectoine) and analyzed their molecular mechanisms. In vitro assays, cellular assays, and skin modeling studies were performed on the samples to provide a reference for the application of D‐Ectoine in cosmetic products. The results showed that D‐Ectoine has a higher T‐AOC and DPPH scavenging activity than ectoine alone at the same concentration. D‐Ectoine promoted GSH‐PX activity and inhibited LPS‐induced inflammatory responses in HSF cells. Furthermore, D‐Ectoine increased cell survival and Nrf2 expression and decreased 8‐OHdG expression after UVB irradiation. Our results supported the potential application of D‐Ectoine in cosmetic products with protection against UVB‐induced photodamage.

## 2 Material and Method

### 2.1 Materials

D‐Ectoine (Cat. No. SC114) was provided from Umotor (Hangzhou, China). WY14643 (C7081), dexamethasone (D4902), and lipopolysaccharide (L2880) were purchased from Sigma‐Aldrich (Shanghai, China). Anti‐Nrf2 antibody (ab62352) and anti‐DNA/RNA damage antibody (ab62623) were purchased from Abcam (Shanghai, China). All other chemicals were from Sigma‐Aldrich or Solarbio (Beijing, China).

### 2.2 DPPH Free Radical Scavenging Assay

The DPPH free radical scavenging capacity kit (Item No. A153‐1‐1) from Nanjing Jiancheng Biological Research Institute was used in this experiment. Working solutions containing 0.1% OAA, 5% D‐Ectoine, and 1% Ectoine prepared as described in the kit were incubated at 25°C for 30 min in darkness and then centrifuged at 4,000 rpm for 5 min to remove precipitate. The absorbance was measured at 517 nm. Each sample was performed in three independent experiments.

(1) $$A=1-\left(A_{\text{assay}}-A_{\text{control}}\right)/A_{\text{blank}}$$

A: Sample DPPH radical scavenging rate (%).

### 2.3 Hydroxyl Radical Scavenging Assay

The hydroxyl radical (OH·^−^) assay kit (Item No. A018‐1‐1) from Nanjing Jiancheng Biological Research Institute was used in this experiment. Working solutions containing 0.1% OAA, 5% D‐Ectoine, and 1% Ectoine prepared as described in the kit were stirred at 37°C for 1 min. Then, 2 mL of colorant was added to terminate the reaction and left at room temperature for 20 min. The absorbance was measured at 550 nm. Each test was performed in three independent experiments.

(2) $$B=\left(A_{\text{control}}-A_{\text{assay}}\right)/\left(A_{\text{standard}}-A_{\text{blank}}\right)\times C_{\text{standard}}\times 1/V_{\text{sample}}\times N$$

C_*standard*_: Standard concentration; V_*sample*_: Sample volume; N: Dilution of the sample before testing; *B*, Hydroxyl radical inhibition capacity (U/ml).

### 2.4 Antisuperoxide Anion Assay

This experiment was performed by using the Inhibition and Generation of Superoxide Anion Free Radical (O_2_·^−^) Assay Kit (Item No. A052‐1‐1) from Nanjing Jiancheng Biological Research Institute. Working solutions containing 0.1% OAA, 5% D‐Ectoine, and 1% Ectoine prepared as described in the kit were incubated at 37°C for 40 min. Then, the 2 mL of colorant was added and left at 25°C for 10 min. The absorbance was measured at 550 nm. Each test was performed in three independent experiments.

(3) $$C=\left(A_{\text{control}}-A_{\text{assay}}\right)/\left(A_{\text{control}}-A_{\text{standard}}\right)\times C_{\text{standard}}\times 1000\times \text{N}$$

C_*standard*_: Standard concentration;

1,000: Unit conversion, ml ⇒ L;

N: Number of times the sample was diluted before testing;

C: Antisuperoxide anion capacity (U/L).

### 2.5 Measurement of Total Antioxidant Capacity

The total antioxidant capacity (T‐AOC) assay kit (Item No. A015‐1) from Nanjing Jianjian Biological Research Institute was used in this experiment. Working solutions containing 0.1% OAA, 5% D‐Ectoine, and 1% Ectoine prepared as desired in the kit were incubated between 25°C and 37°C for 10 min. The absorbance was measured at 520 nm. Each test was performed in three independent experiments.

(4) $$D=\left(A_{\text{assay}}-A_{\text{control}}\right)/0.01/T\times V_{\text{total reaction}}/V_{\text{sample}}\times N.$$

T: Reaction time, 30 min; V_*total reaction*_: Total volume of reaction system, ml; V_*sample*_: Sample volume, ml; N: Sample pretest dilution; D: Total antioxidant capacity (U/ml).

### 2.6 MTT Assay

HSF cells in the logarithmic phase were collected and prepared for suspension with 1640 complete medium at 2 × 10^5^ cells/ml. 100 μL of cell was added in 96‐well plate and incubated at 37°C. Then, the cells were, respectively, treated with D‐Ectoine(0, 1%, 2.5%, 5%, and 10%) for a 24 h‐incubation. After washed with 200 μL of PBS solution (pH 6.8) for two times. 200 μL of medium and 20 μL of MTT solution were added to each well and incubated for 3 h. The medium was carefully aspirated from the wells, and 100 μL of dimethyl sulfoxide (DMSO) was added and shaken for 10 min. The absorbance was measured at 490 nm, and the cell survival rate was calculated. A cell survival rate greater than 85% indicates no side‐effect on cell proliferation at that concentration, which was considered the cell safety concentration. Each test was performed in six independent experiments.

### 2.7 Assay of GSH‐PX, CAT, SOD, and MDA Content

The HSF cells culture method was the same as 2.5 until the old medium was removed, and then control, 0.1% 3OAA and 5% D‐Ectoine were added to the medium, respectively. Cells were spiked and placed in a 37°C for 48 h. The cells and culture medium were ultrasonically broken for 8 min (each time for 8 s with an interval of 5 s). After that, centrifugation was performed at 1000 × g for 10 min, and the supernatant was tested. The detection kits of GSH‐PX (A005‐1), CAT (A007‐1‐1), SOD (A001‐3), and MDA (A003‐4‐1) from Nanjing Jiancheng Biological Research Institute were used in the samples tests. Each test was performed in three independent experiments.

### 2.8 Measurement of Inflammatory Factors IL‐6 and IL‐8

The HSF cells culture method was the same as 2.5 until the old medium was removed, and then 2 μg/mL lipopolysaccharide(LPS), 0.625 μg/mL(1.59 μM) dexamethasone (DXM), and 5% D‐Ectoine were added to the medium as inducer for inflammatory factors, positive control, and test sample, respectively. The cells were incubated at 37°C for 24 h, and supernatant was tested. IL‐6 and IL‐8 were detected using the corresponding ELISA kits, which included ab178013 and ab214030 from Abcam. Each test was performed in three independent experiments.

### 2.9 Assay of Col I and MMP‐1 Content

The HSF cells culture method was the same as 2.5 until the old medium was removed, and then 100 ng/mL TGF‐β1 and 5% D‐Ectoine were added to the medium, respectively. The cells were cultured at 37°C for 24 h with and without UVB (600 mJ/cm^2^). Then, the cell supernatant was collected and tested. Human Col I ELISA Kit (CSB‐E08082h, Cusabio) and MMP‐1 ELISA kit (KE00223, Proteintech) were utilized to detect the content of Col I and MMP‐1 in the samples. Each test was performed in three independent experiments.

### 2.10 Culture, Administration, Irradiation and Detection of Skin Models

The 3D epidermal skin model (EpiKutis) and the EpiGrowth culture medium were provided by Guangdong Boxi. The model was randomly divided into the blank control group (BC), negative control group (NC), positive control group (PC), and sample group in a six‐well plate. The BC and NC groups only included medium. The PC group included equal amounts of culture medium containing 50 μM WY14643, 7 μg/mL VE, and the sample group included 5% D‐Ectoine. All groups, except for the blank control group, were irradiated with UVB at a dose of 600 mJ/cm^2^. After irradiation, the six‐well plates were incubated at 37°C with 5% CO_2_ for 24 h, and the surface of the model was cleaned with sterile PBS. For the histomorphometric assay, the models were fixed with 4% paraformaldehyde for 24 h and were subjected to H&E staining or immunofluorescence assay. Images were taken under a microscope for observation and subsequently captured and analyzed. Each test was performed in three independent experiments.

### 2.11 Statistical Analysis

The experiments were performed in multiple replicates, and data were expressed as the mean ± SEM. The statistical test used in data analysis to calculate statistical significance was indicated in respective figure legends. The control ingredients in the experiments were listed in Table 1.

**TABLE 1:** Positive and negative control ingredients used in this paper.

Ingredients	Dose	Stimulants	Action mode
3OAA	0.1%–1%	/	Antioxidant
DXM	0.625 μg/mL (1.59 μM)	LPS	Inflammatory inhibitor
TGF‐β1	100 ng/ml	UVB	ECM driver
WY‐14643	50 μM	UVB	Cells protector
VE	7 μg/ml	UVB	Antioxdiant

## 3 Results

### 3.1 D‐Ectoine has Enhanced Antioxidant Activity at the Molecular Level

D‐Ectoine came from Ectoine– *T. thermophilus* cofermentation products, which contain 20% Ectoine and *T. thermophilus* cell lysates. We named it D‐Ectoine in that D could mean doubled or developed for the effects of D‐Ectoine compared to ectoine.

**FIGURE 1:**
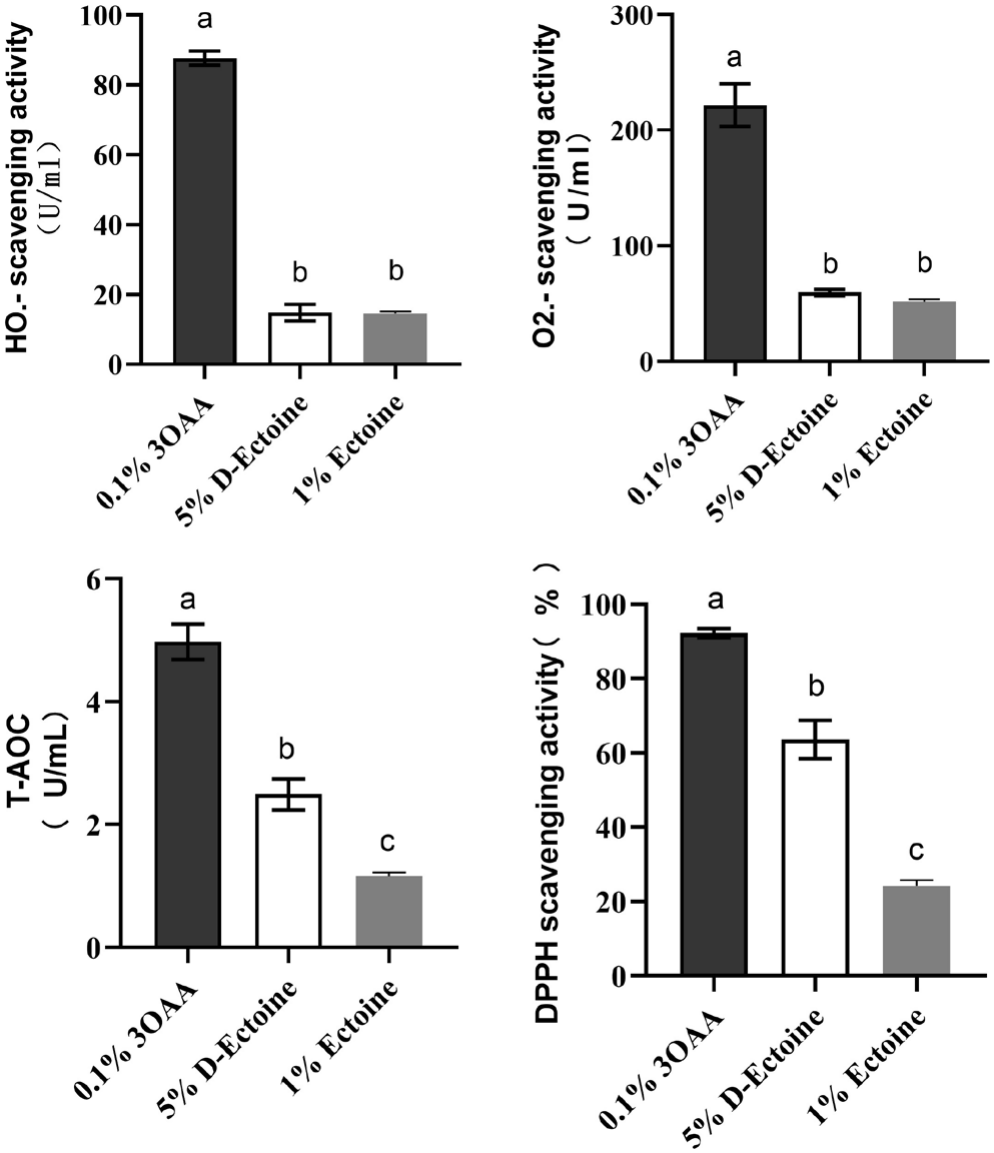
The antioxidant activities comparison between 5% D‐Ectoine and 1% Ectoine. 0.1% 3OAA was a positive control. Results were subjected to ANOVA, and *p* < 0.05 was regarded as significant.

Excessive free radicals could attack biological macromolecules, such as cell membranes and proteins, and result in wrinkle formation, elasticity loss, and collagen degradation in the skin. Antioxidant active ingredients can scavenge free radicals, reduce harmful skin issues, and slow down skin aging. To study the D‐Ectoine antioxidant activity, solutions containing 5% D‐Ectoine (which includes 20% ectoine) and 1% ectoine were used to analyze their antioxidant activity in OH·^−^ scavenging assay, O_2_·^−^ assay, T‐AOC assay, and DPPH scavenging assay. Antioxidant 3‐O‐ethyl ascorbic acid (3OAA) was added as a positive control [31]. The results showed that there was no significant difference in OH·^−^ scavenging capacity and O_2_.^−^ scavenging capacity between D‐Ectoine and ectoine. The T‐AOC and DPPH scavenging capacity of D‐Ectoine was significantly better than that of ectoine. This suggests that D‐Ectoine, containing *T. thermophilus* fermentation products, has superior antioxidant capacity to ectoine alone (Figure 1).

### 3.2 Antioxidant Activities of 5% D‐Ectoine In Vitro

ROS can attack polyunsaturated fatty acid (PUFA) in biological membranes, triggering lipid peroxidation and the formation of lipid peroxides, resulting in the production of various byproducts, including aldehydes such as MDA, hydroxyl groups, and new oxygen radicals. SOD is an important antioxidant and catalyzes the conversion of superoxide anion (O^2−^) into H_2_O_2_ and O_2_. CAT, as a marker enzyme of peroxisome, catalyzes the decomposition of hydrogen peroxide into oxygen and water. GSH‐PX promotes the reaction of H_2_O_2_ with reduced glutathione (GSH) to form H_2_O and oxidized glutathione (GSSG). The level of SOD, CAT, and GSH‐PX activity reflects the ability to scavenge ROS, while the level of MDA reflects the severity of ROS in cells.

**FIGURE 2:**
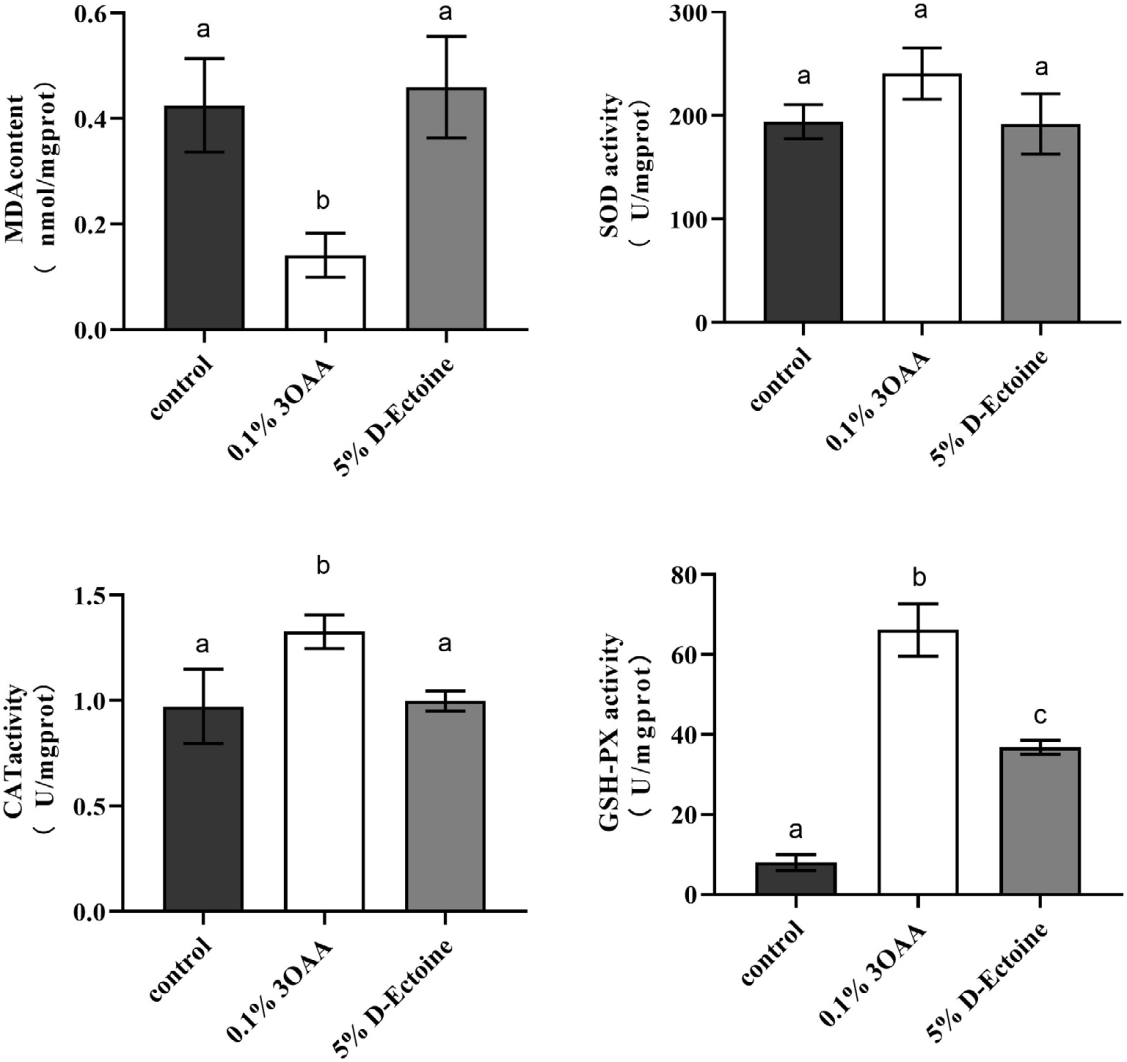
The cellular antioxidant activities of D‐Ectoine. 0.1% 3OAA was a positive control. Results were subjected to ANOVA, and *p* < 0.05 was regarded as significant.

To investigate the antioxidant effect of the D‐Ectoine in cells, we observed the MDA content, SOD activity, CAT activity, and GSH‐PX activity in the HSF cells after being treated with 5% D‐Ectoine. It was found that the MDA content, SOD activity, and CAT content in the fibroblasts treated with 5% D‐Ectoine were not significantly different from those of the control group. However, the GSH‐PX activity was significantly enhanced compared with that of the control group. It indicates that 5% D‐Ectoine can increase the intracellular GSH‐PX activity, thus improving the antioxidant capacity of the cells (Figure 2).

### 3.3 Effects of D‐Ectoine on the Viability of HSF Cells

**FIGURE 3:**
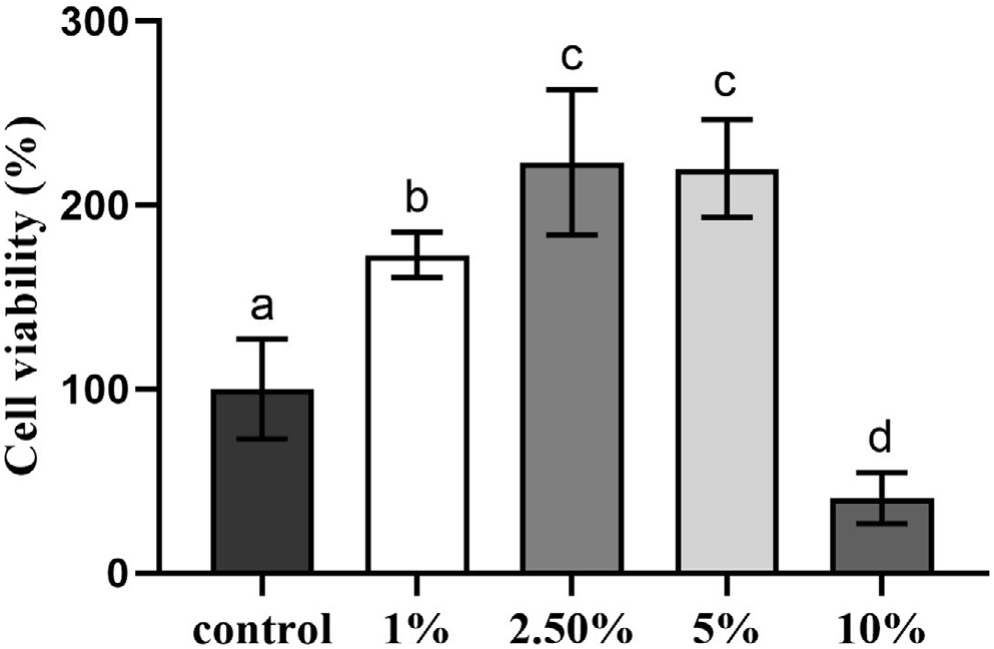
Effects of D‐Ectoine on viability in fibroblast cells. Results were subjected to ANOVA, and *p* < 0.05 was regarded as significant.

The cytotoxic character of D‐Ectoine was examined by MTT assay. As shown in Figure 3, cell viability increases significantly with control when concentration is lower than 5%. This indicates that D‐Ectoine has no toxic effect on fibroblasts and stimulates cell proliferation at an appropriate concentration.

### 3.4 D‐Ectoine Mitigates LPS‐Induced Inflammatory Response

**FIGURE 4:**
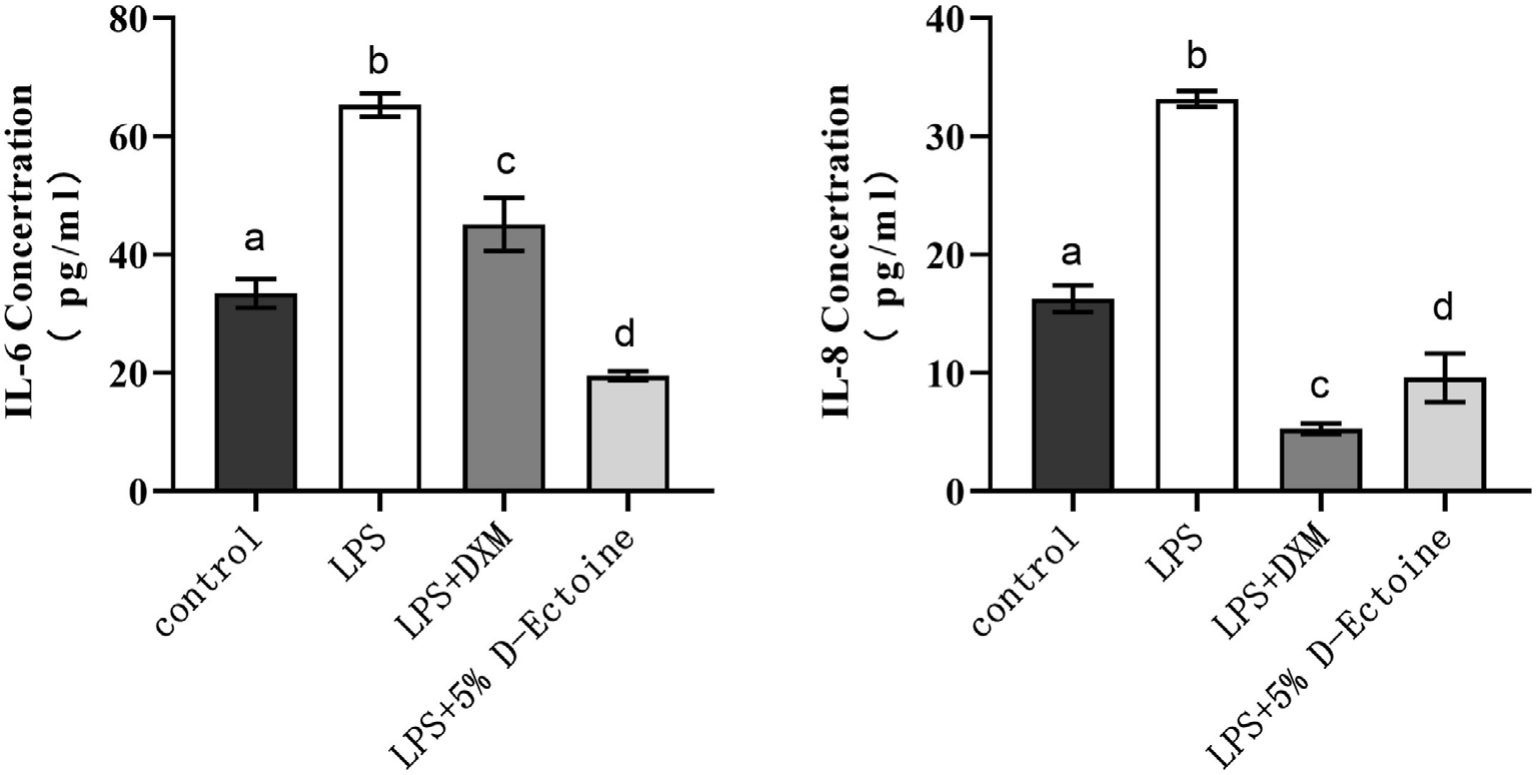
Effects of D‐Ectoine on LPS‐induced inflammation in fibroblast cells. Results were subjected to ANOVA, and *p* < 0.05 were regarded as significant.

Since the production of ROS might cause an inflammatory response, to assess the degree of skin inflammation, LPS is used to stimulate HSF cells, thereby inducing an inflammatory response [32]. The IL‐6 and IL‐8 expression levels were significantly upregulated by 2 μg/mL LPS, while the positive control drug 0.625 μg/mL (1.59 μM) dexamethasone (DXM) and 5% D‐Ectoine inhibited these LPS‐induced increases in inflammatory factor expression. Together, these data confirmed that 5% D‐Ectoine could effectively reduce inflammatory responses (Figure 4).

### 3.5 D‐Ectoine Induced Collagen‐1 Expression in HSF Cells

Type I collagen (Col I) is a primary constituent of the ECM in skin, bones, and connective tissues. In the skin, Col I is the most abundant component in the dermal connective tissue, accounting for more than 80%. It provides support and elasticity for the skin. As collagen is lost, the skin loses its elasticity and firmness, leading to skin aging. Thus, human fibroblasts can be used as a cell model to study the expression change of type I collagen content, and transforming growth factor beta1 (TGF‐β1) was used as the positive control [33].

**FIGURE 5:**
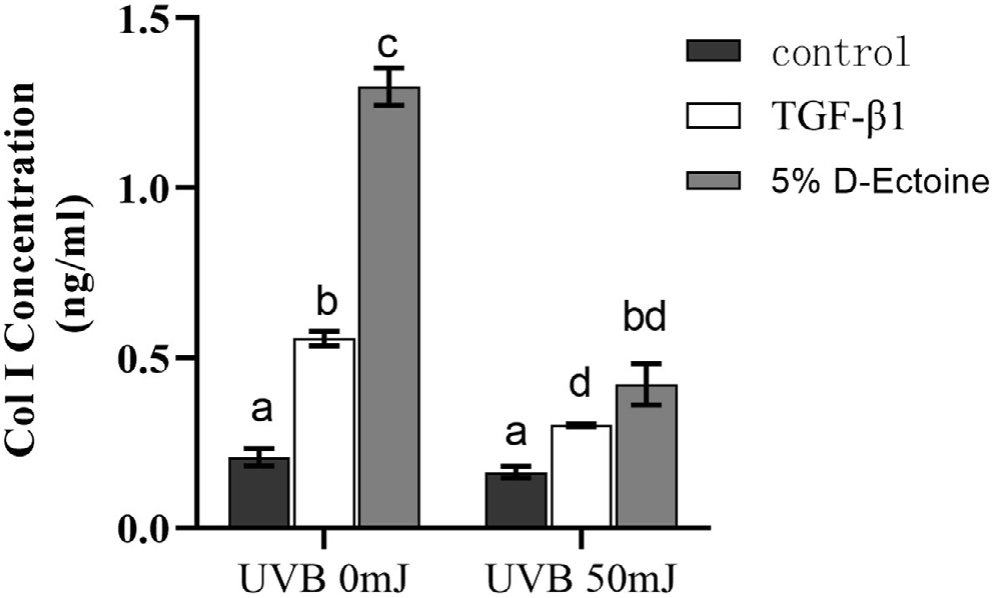
Intracellular collagen I content after UVB induction in fibroblast cells. Results were subjected to ANOVA, and *p* < 0.05 were regarded as significant.

It can be found that the expression of Col I increased significantly after treatment with 100 ng/mL TGF‐β1 and 5% D‐Ectoine under normal culture conditions without UVB irradiation. At the same time, compared to the positive control, the elevation of expression was more obvious after treatment with 5% D‐Ectoine, indicating that 5% D‐Ectoine had the effect of promoting collagen type I expression. After UVB irradiation, the expression of Col I increased significantly after treatment with TGF‐β1 and 5% D‐Ectoine compared to the control group. However, the elevated level of Col I expression was reduced compared to cells not exposed to UVB irradiation. This indicates that although the UVB irradiation inhibited the Col I expression in HSF, TGF‐β1 and 5% D‐Ectoine still retain the ability to promote Col I expression (Figure 5).

### 3.6 D‐Ectoine Inhibited the UVB‐Induced MMP‐1 Expression in HSF Cells

MMP‐1 is a member of the matrix MMP family. MMPs are a highly conserved group of proteases that are responsible for degrading nearly all components of the ECM, and overexpression of MMP‐1 leads to the specific degradation of ECM components, disrupting the normal structure of collagen and elastin fibers, which can result in skin wrinkles and other signs of aging. Based on the important role of MMP‐1 in the skin aging process, inhibition of the MMP‐1 enzyme has been gradually used as a new way to delay aging. Human dermal fibroblasts can serve as a cellular model to investigate the inhibitory effects of cosmetics on MMP‐1.

**FIGURE 6:**
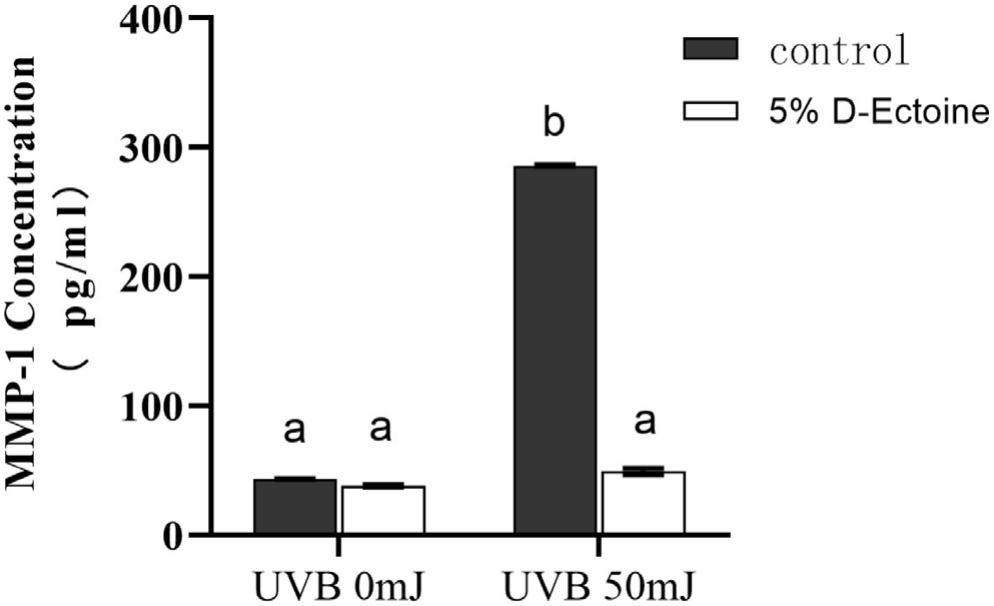
Intracellular MMP‐1 content after UVB induction in fibroblast cells. Results were subjected to ANOVA, and *p* < 0.05 was regarded as significant.

It could be measured that there was no significant difference in the expression of MMP‐1 after treatment of the cells with 5% D‐Ectoine compared with the control under normal culture conditions without UVB irradiation. However, after UVB irradiation, the expression of MMP‐1 in HSF cells was significantly upregulated compared to that in non‐irradiated HSF cells. In contrast, HSF cells treated with 5% D‐Ectoine showed significantly lower MMP‐1 expression than the UVB‐irradiated control group, suggesting that 5% D‐Ectoine has an inhibitory effect on HSF MMP‐1 expression under UVB irradiation conditions (Figure 6).

### 3.7 D‐Ectoine Significantly Reduced UVB‐Induced Cell Damage in EpiKutis

The 3D epidermal model is widely used for efficacy testing of skin barrier, moisturizing, anti‐inflammatory, UV protection, and transdermal absorption in cosmetics and pharmaceutical studies. In our test, a UVB‐irradiated 3D epidermal skin model (EpiKutis) was used to determine the protective effects of samples on the skin by examining tissue and cell morphology through hematoxylin and eosin (H&E) staining.

**FIGURE 7:**
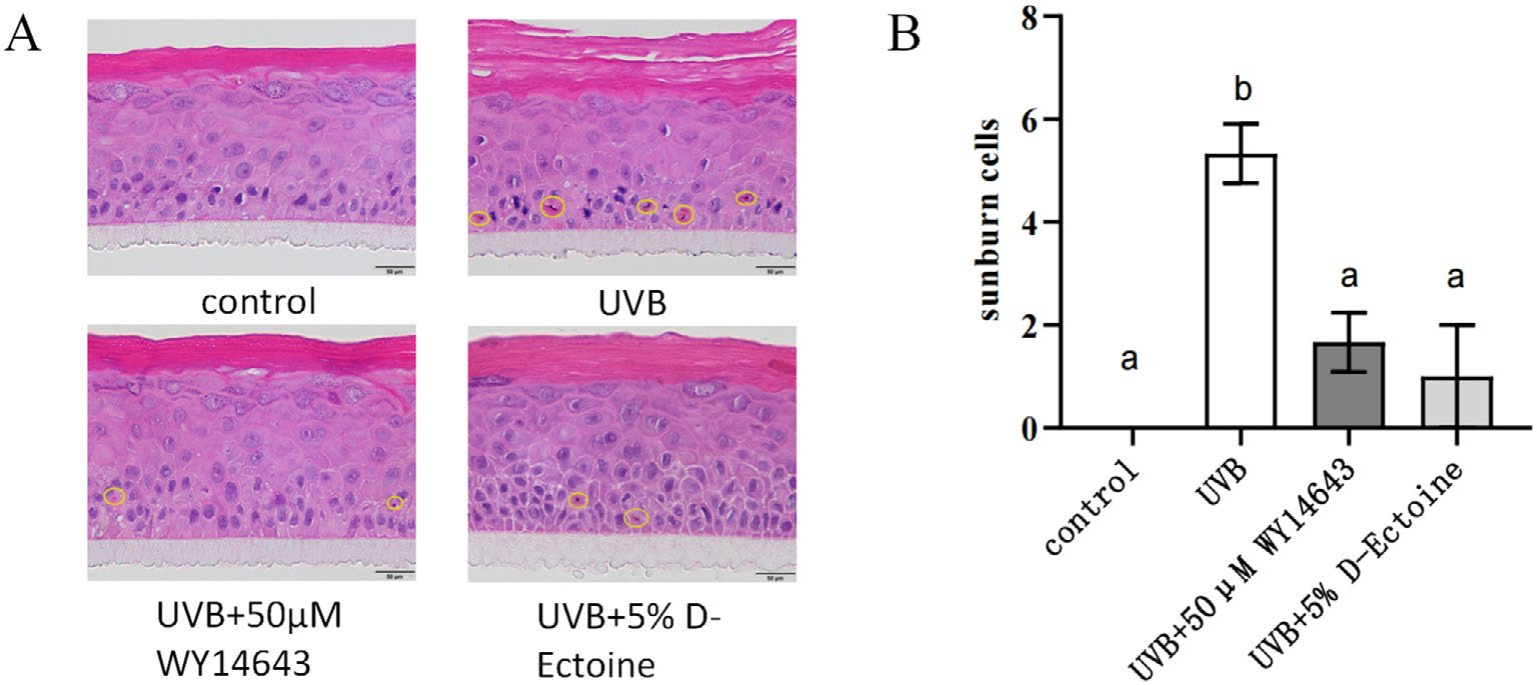
Histopathological assessment of cell injury after UVB induction in EpiKutis. A, Representative images of sunburn cells induced by UVB. WY14643 was used as a positive control. Yellow circles show condensed nucleus. Bars indicate 50μm in length. B, Quantitation of sunburn cells in EpiKutis induced by UVB. n=3. Results were subjected to ANOVA, and *p* < 0.05 was regarded as significant.

Under normal conditions, H&E‐stained cells exhibited a healthy morphology with rounded and full nuclei. However, after UVB irradiation, some cell nuclei displayed signs of damage, such as wrinkling, indicating cellular damage due to UVB exposure. WY14643 is an effective PPARα agonist known for its protective effects on damaged cells [34]. The morphology of nucleus is condensed after UV irradiation [35]. The number of abnormal nucleus was counted. A reduction in the number of UVB‐damaged cells in WY14643‐treated cells suggested that WY14643 could protect cells from UVB damage. Similarly, a significant reduction in damaged cells was observed in 5% D‐Ectoine treated cells, indicating that D‐Ectoine also possesses the ability to protect cells from UVB‐induced skin damage (Figure 7).

### 3.8 D‐Ectoine Attenuated UVB‐Induced Reduction of Nrf2 Protein Abundance in EpiKutis

Nrf2 is a crucial transcription factor that regulates the cellular response to oxidative stress and plays a central role in maintaining intracellular redox homeostasis. By inducing and modulating the expression of a range of antioxidants, such as vitamin E (VE) [36], Nrf2 can mitigate cellular damage caused by ROS and electrophilic compounds, thereby preserving cellular stability and upholding the body's redox balance. The expression levels of specific proteins within cells and tissues can be visualized and examined through immunohistochemistry staining. In addition, the immunohistochemistry staining integrated optical density (IOD) value is a parameter used to quantify the expression level of a target protein by measuring optical density.

**FIGURE 8:**
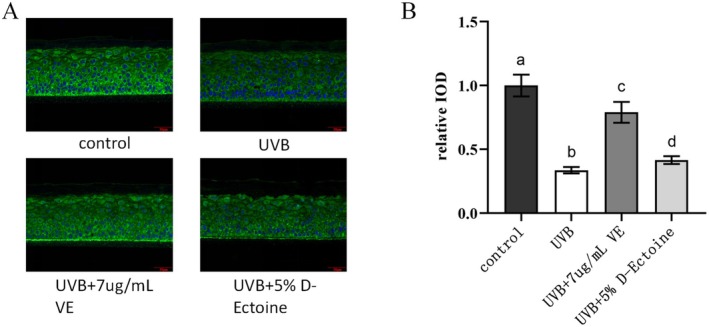
D‐Ectione on UVB‐induced Nrf2 signaling in EpiKutis. A, Immunohistochemistry staining images of NRF2 induced by UVB. VE was used as a positive control. Green staining indicated NRF2, and blue DAPI staining indicated nuclei. Bars indicate 50μm in length. B, Relative IOD of NRF2 in EpiKutis induced by UVB. n=3. Results were subjected to ANOVA, and *p* < 0.05 was regarded as significant.

Data analysis showed that the relative IOD values decreased significantly after UVB irradiation, indicating that UVB irradiation reduced the protein expression level of Nrf2. The IOD values treated with 7 μg/mL of VE were significantly higher than those of the UVB‐irradiated group, suggesting that VE has a protective effect on Nrf2 protein expression. The relative IOD values treated with 5% D‐Ectoine were slightly higher compared to the UVB‐irradiated group, indicating that 5% D‐Ectoine could modestly increase the protein expression level of Nrf2 under conditions of UVB irradiation (Figure 8).

### 3.9 D‐Ectoine Reduced UVB‐Induced DNA Damage in EpiKutis

OHdG is an oxidative DNA adduct that forms when ROS, such as hydroxyl radicals and singlet oxygen, attack the 8th carbon atom of the guanine base in DNA molecules. 8‐OHDG is used as a biomarker for the oxidative damage of DNA caused by both endogenous and exogenous factors, and it can be used to assess the degree of oxidative damage and repair in vivo.

**FIGURE 9:**
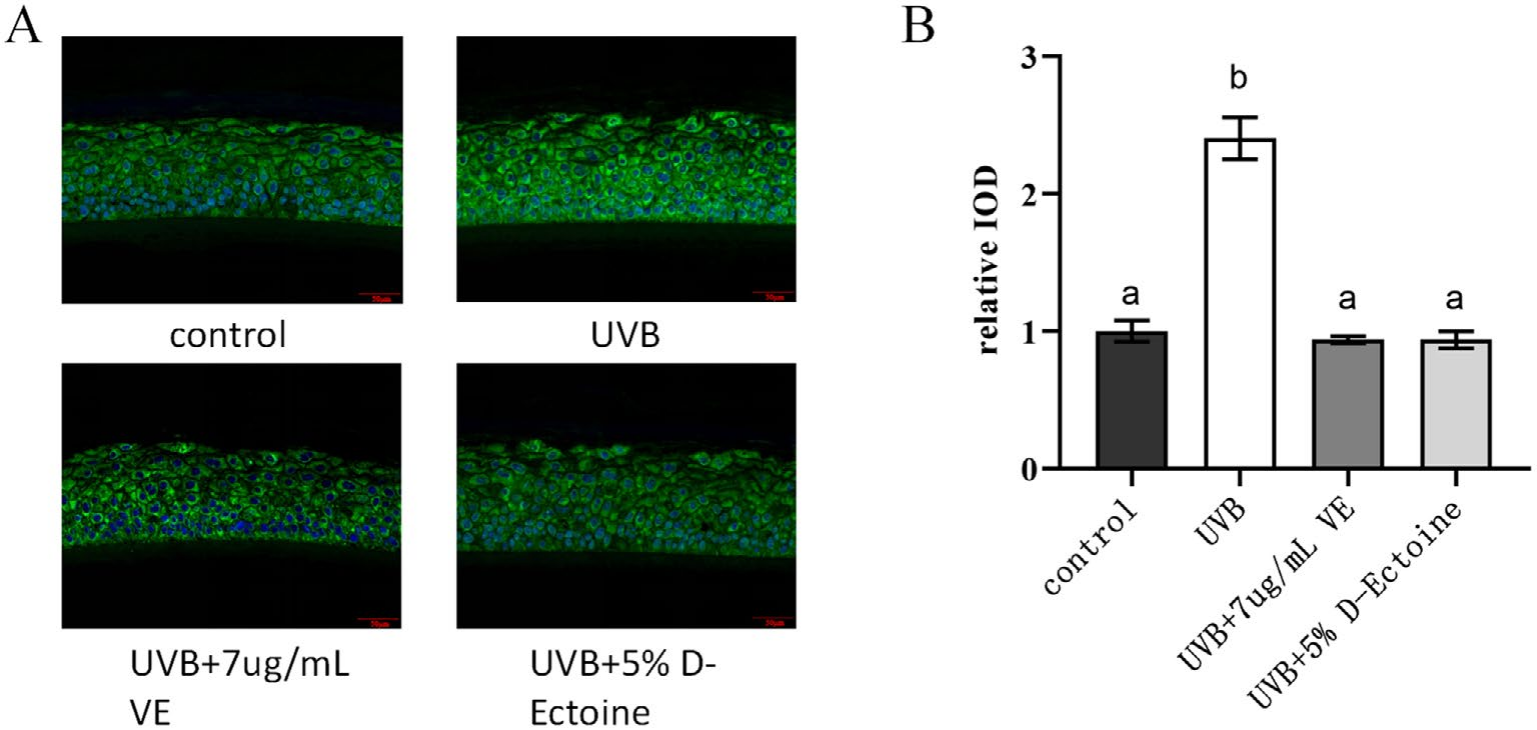
D‐Ectione on UVB‐induced 8‐OHdG expression in EpiKutis. A, Immunohistochemistry staining images of 8‐OHdG induced by UVB. VE was used as a positive control. Green staining indicated 8‐OHdG, and blue DAPI staining indicated nuclei. Bars indicate 50μm in length. B, Relative IOD of 8‐OHdG in EpiKutis induced by UVB. n=3. Results were subjected to ANOVA, and *p* < 0.05 was regarded as significant.

Data analysis showed that the relative IOD value significantly increased after UVB irradiation, indicating that UVB exposure elevated the levels of 8‐OHdG, resulting in DNA damage. The IOD value treated with 7 μg/mL Vitamin E (VE) decreased significantly, suggesting that VE inhibited 8‐OHdG expression. The relative IOD values in the 5% D‐Ectoine treated skin model also decreased significantly compared to the UVB‐irradiated group, indicating that 5% D‐Ectoine could significantly reduce the expression level of 8‐OHdG (Figure 9).

## 4 Discussion

Our results showed that the T‐AOC and DPPH scavenging capacity of D‐Ectoine were significantly better than that of ectoine alone, suggesting that the fermentation products of *T. thermophillu* enhanced the antioxidant capacity of ectoine. Further study on the antioxidant, anti‐inflammatory, antidegenerative, and UVB protection abilities of D‐Ectoineshowed that D‐Ectoine could increase the activity of GSH‐PX in fibroblasts and reduce the expression of LPS‐induced inflammatory factors IL‐6 and IL‐8. D‐Ectoine significantly developed Col I expression in both control and UVB irradiation, but reduced the expression of MMP‐1, particularly under UVB irradiation. It appears that there are more collagen proteins participating. In EpiKutis, D‐Ectoine promoted cell survival, increased the expression of Nrf2, and inhibited the expression of 8‐OHdG under UVB irradiation. These findings suggest that D‐Ectoine plays a significant role in combating skin photoaging and is a promising cosmetic product.

Ectoine is a valuable resource provided by halophilic microorganisms and has been widely used in cosmetics for its ability to stabilize proteins, nucleic acids, biofilms, and whole cells [37]. *T. thermophilus* ferment, which includes amino acids, peptides, and thermostable enzymes, is also derived from extreme environment microorganisms. In this study, we used ectoine combined with the *T. thermophilus* ferment to obtain D‐Ectoine samples and investigated its efficacy and molecular mechanisms in vitro and in cells.

Primary and secondary metabolites from microbial fermentation have found numerous applications across various industries, including pharmaceuticals, food, energy, chemicals, and agriculture, as well as biological nitrogen fixation and engineered biopesticides [38]. Among them, extreme environmental microorganisms and their products have received extensive attention in recent years. For example, thermophilic enzymes and barophilic enzymes from thermophiles and piezophiles are extremely stable and can be used to process food under aseptic conditions [39]. They can be adapted to a wide range of physicochemical conditions and can also be used in a variety of industrial processes such as textiles, leather, cosmetics, or pharmaceuticals. Due to their unique properties, a large number of interactions and symbiotic relationships can modulate the dynamics of bacterial and archaeal communities in extreme environments. Therefore, these microorganisms are an unexplored treasure trove of genetic resources. Consequently, research focused on both the microbial diversity of extreme environments and its industrial applications is of immense significance.

## 5 Conclusion

The antiphotodamage properties of D‐Ectoine include higher antioxidant activity, protection with antioxidant enzymes, inhibition of inflammatory response and MMP‐1 content, as well as improving cell survival and Nrf2 expression, decreasing DNA damage. This study serves as a new theoretical basis for potential antioxidant drugs to treat skin photodamage, and D‐Ectoine may also become a topically potential cosmetics agent related to oxidative stress and photodamage.

## Author Contributions

Y. Wang and J. Liang performed the inflammatory factors analysis and all UVB irradiation experiments, P. Zhang and G. Yan provided D‐Ectoine sample and its antioxidant activity, H. Jiang and M. Wang designed the experiments and analyzed the data, Y. Wang, J. Liang, and M. Wang wrote the manuscript. All authors have read and agreed to the published version of the manuscript.

## Conflicts of Interest

The authors declare no conflicts of interest.

## Data Availability

The data that support the findings of this study are available from the corresponding author upon reasonable request.
